# Research hotspots and frontiers of acceptance and commitment therapy for mental disorders: a bibliometric analysis

**DOI:** 10.3389/fpsyt.2025.1440755

**Published:** 2025-06-18

**Authors:** Xueqing Wang, Mingqi Wang, Huafang Li

**Affiliations:** ^1^ School of Nursing, Shandong Second Medical University, Weifang, China; ^2^ School of Physical Education, Shandong University, Jinan, China; ^3^ Department of Rehabilitation Medicine, Shandong Mental Health Center, Shandong University, Jinan, China

**Keywords:** acceptance and commitment therapy, mental disorders, hotspots, CiteSpace, bibliometric analysis

## Abstract

**Background:**

Mental disorders represent a complex and increasingly prevalent group of chronic illnesses, with a prevalence that is increasing significantly. Acceptance and Commitment Therapy (ACT), as an important branch of psychotherapy, is receiving increasing attention. This study aims to provide a comprehensive overview of current research status, hotspots, and future development trends of ACT for mental disorders.

**Methods:**

A search was conducted in the Web of Science Core Collection to identify English-language publications on ACT for mental disorders from 1 January 2010 to 1 March 2024. The CiteSpace software was employed to analyze the retrieved records, including countries, institutions, journals, authors, as well as a keyword co-occurrence analysis.

**Results:**

790 publications on ACT for mental disorders were analyzed in this study, comprising 665 articles and 125 reviews. Since 2010, the number of publications has demonstrated a consistent upward trajectory on an annual basis. The most prolific author was Twohig MP from Utah State University, with 32 publications. The author with the highest average citation frequency is Bohlmeijer ET, with an average of 106.21 citations per paper. The highest number of publications were published in the “Journal of Contextual Behavioral Science” (94 papers), with an impact factor of 5.0. The most productive countries and institutions were the United States (283 papers) and Karolinska Inst (47 papers), respectively. A keyword analysis revealed that the most frequently occurring keywords were “acceptance and commitment therapy”, “depression”, and “cognitive behavioral therapy”.

**Conclusions:**

The number of studies on ACT for mental disorders has demonstrated a consistently increasing trend from year to year. In the clinical context, ACT is regarded as a promising method for improving mental health and psychiatric symptoms in patients with mental disorders. The visualization analysis can facilitate researchers in gaining a deeper understanding of the current status and development trend of research in this field, thereby encouraging more researchers to devote attention to the application of ACT in mental disorders.

## 1 Introduction

Mental disorders are a group of complex and persistent chronic illnesses that usually result in significant cognitive, emotional, volitional and behavioral abnormalities (1). Mental disorders cover a wide range of types including, but not limited to, anxiety disorders, depressive disorders, obsessive-compulsive disorders(OCD), schizophrenia, and substance-related disorders (2). The World Health Organization (WHO) has estimated that the global prevalence of mental disorders stands at approximately one billion individuals (3). It is projected that mental disorders will be the leading cause of the global disease burden by 2030 (4). A number of studies have demonstrated that individuals with mental disorders have a life expectancy that is 10–15 years shorter than that of the general population (5). Moreover, the prevalence of chronic physical diseases and the risk of premature mortality are significantly higher in patients suffer from mental illness (6–8). Due to the high prevalence, low cure rates, and long treatment course (9), mental illness has a profound negative impact on individuals’ activities of daily living, social functioning, and quality of life. Therefore, further research and the development of effective treatments for mental disorders are of particular urgency and importance.

Medication therapy is currently considered the first-line treatment for psychiatric disorders and has demonstrated good therapeutic efficacy (10). However, there is mounting evidence to suggest that approximately 30% to 40% of patients with psychiatric disorders do not respond well to the antidepressants (11). Long-term use of medication may also result in a number of adverse effects, including metabolic syndrome, gastrointestinal dysfunction, and sexual dysfunction. These adverse effects significantly reduce patients’ adherence to treatment and increase the risk of relapse (12, 13). Psychotherapy, as one of the representative therapeutic means of non-pharmacological treatment, has shown promising applications in the treatment of mental disorders (14). Psychotherapy refers to the use of professional psychological techniques and theories by the therapist to establish a unique therapeutic relationship with the patient. The aim of psychotherapy is typically to assist patients in resolving psychological distress, improving emotional state, enhancing cognitive abilities and social adaptability (15).

ACT has emerged as a significant branch of psychotherapy, garnering considerable interest in the field of mental disorders in recent years. The approach was established in the 1990s by Steven C. Hayes and other professors of psychology at the University of Nevada, USA, based on Cognitive Behavioral Therapy (CBT) (16). ACT is considered one of the most representative empirical behavioral psychotherapies in the “third wave” of CBT (17). The core therapeutic processes of ACT include the following: acceptance, cognitive dissociation, being present, the self as context, values clarification, and committed action (18). Therefore, this method aims to fundamentally alter the relationship between patients and their symptoms. This is achieved by encouraging patients to accept the inevitable pain and distress in their lives, thereby enabling them to live full, rich, and meaningful lives (12). Over the past decade, a large number of studies have been conducted to investigate effects and mechanisms of ACT in psychiatric disorders, including anxiety disorders (19), depressive disorders (20), and OCD (21). A systematic review conducted by Kelson et al. (19) demonstrated that web-based ACT is an effective and accessible treatment for patients with anxiety disorders, significantly improving the anxiety symptoms. A qualitative study by Kaipainen et al. (20) found that nurses reported through focus group interviews that mobile applications based on ACT can effectively assist in the care of depression and other mental health issues. A meta-analysis (21) demonstrated that ACT has a positive effect on obsessive-compulsive symptoms in patients with OCD. Additionally, it can be an effective complement to other available treatments. Additionally, several studies have indicated that ACT may also have potential effects for psychiatric disorders such as post-traumatic stress disorder, schizophrenia and substance-related disorders (22–24). In light of the promising developments of ACT for mental disorders, it is necessary to conduct a systematic bibliometric analysis of the relevant literature. This will provide researchers with a comprehensive overview of the current research status, research hotspots, and development trends in ACT for mental disorders.

Bibliometrics, as a systematic and quantitative research method, is frequently employed to analyze scientific literature in a specific field, aiming to identify current research hotspots and trends (25, 26). Since its inception in 1969 by Allen Pritchard (16), a renowned British informatician, bibliometric analysis has evolved into a pivotal methodology for interdisciplinary research, with a pervasive presence across diverse research domains, including psychology, neurology, and pharmacological ecology (16, 27, 28). CiteSpace software is a well-recognized tool for bibliometric analysis, developed by Dr. Chaomei Chen (29). The software’s powerful functionality in visualization analysis provides researchers with an effective way for attaining a more profound comprehension of the current status and hotspots within the research domain. Analysis using CiteSpace software enables researchers to gain a comprehensive understanding of the research dynamics in a particular field, thereby providing theoretical references for future research directions. Despite the increasing research on ACT for mental disorders, there is a lack of comprehensive bibliometric analyses that systematically examine the current status, hotspots, and emerging trends in ACT for mental disorders. Consequently, the objective of this study is to conduct a comprehensive analysis of ACT for mental disorders using CiteSpace software. This analysis will assess the academic value of the study through citation analysis, journals, impact factor, and other metrics, with the aim of providing theoretical guidance for subsequent research.

## 2 Methods

### 2.1 Data collection and search strategy

A comprehensive search strategy was employed to identify all literature on ACT for mental disorders listed in the WOSSC database. The literature was retrieved by utilizing the following search terms: Topic=(“Mental Disorder” OR “Psychiatric illness” OR “Mental illness” OR “Anxiety Disorders” OR depression OR insomnia OR somnia OR “Impulse Control Disorders” OR “Dissociative Disorders” OR “Elimination Disorders” OR “Feeding and Eating Disorders” OR “Motor Disorders” OR “Bipolar and Related Disorders” OR “Depressive Disorder” OR “Bipolar Disorder” OR “Motor Disorders” OR “Neurodevelopmental Disorders” OR “Neurocognitive Disorders” OR “Paraphilic Disorders” OR Schizophrenia OR “Personality Disorders” OR “Sexual Dysfunctions” OR “Sleep Wake Disorders” OR “Somatoform Disorders” OR “Substance-Related Disorders” OR “Trauma and Stressor Related Disorders”) AND Topic=(“Acceptance and Commitment therapy” OR “Commitment and acceptance method” OR “commitment acceptance therapy”). In order to ensure that the retrieved data is both in-depth and comprehensive, it is necessary to select the “Topic” field (title, abstract, or keywords). The search period was limited to the interval between 1 January 2010 and 1 March 2024. The publications included were limited to articles and reviews, and the language was restricted to English. In order to ensure that the included literature was relevant to the topic of this study, two researchers independently retrieved data from the WOSCC database and read the titles and abstracts of all the literature separately. A total of 1,085 documents were retrieved based on the aforementioned search criteria. Of these, 295 were excluded from the analysis as they did not align with the study topic. This resulted in the inclusion of 790 documents for analysis, comprising 665 articles and 125 reviews. Upon completion of the literature screening, all literature information was exported to a “plain text” file, and “full record and citation reference” format for subsequent data processing and analysis. The following key information was extracted from the included literature: title, author, year of publication, keywords, journal, institution, country/regions, type of literature, and number of citations. This will provide an important basis for subsequent data analysis and research.

### 2.2 Data analysis

**Figure 1:**
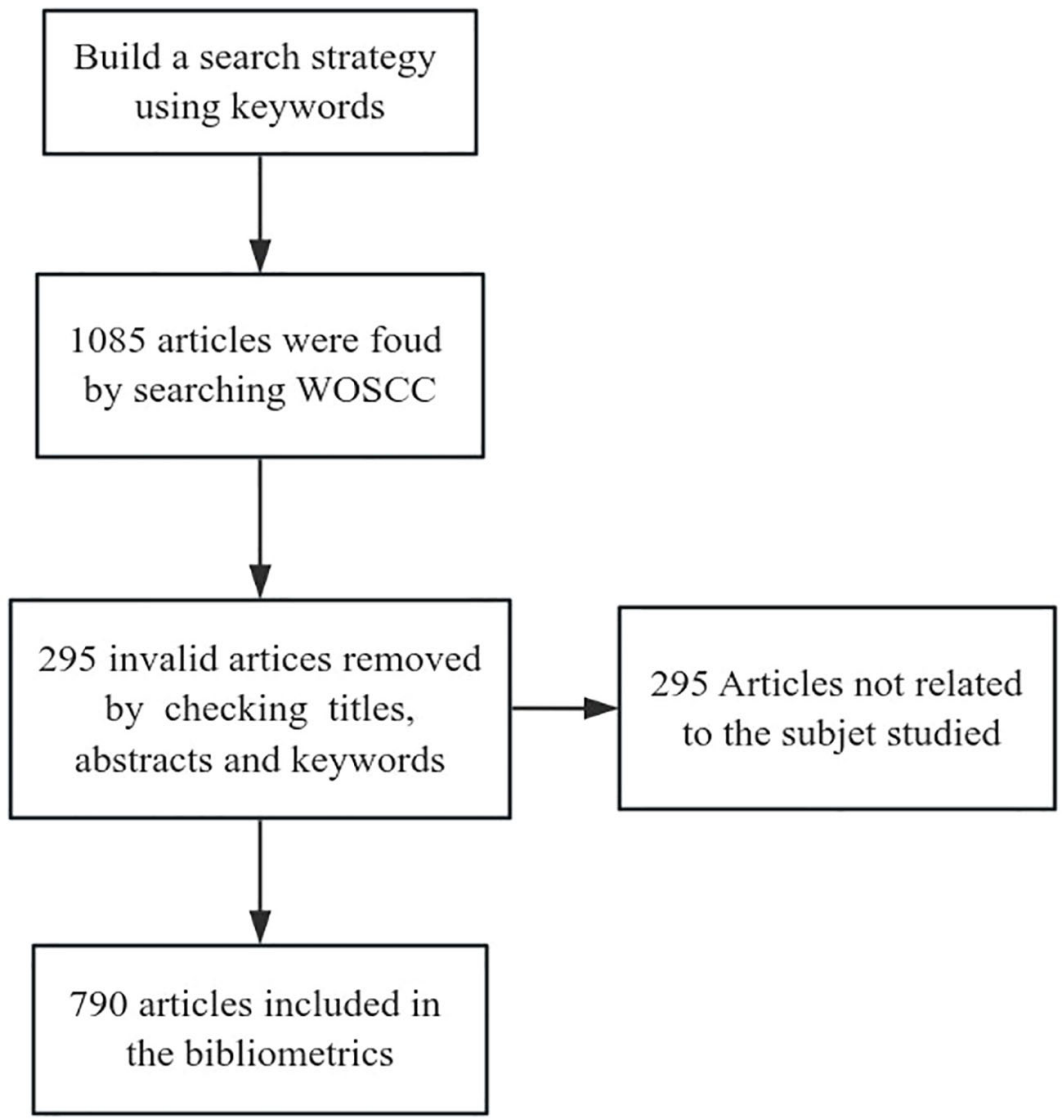
Data search and selection process.

Bibliometric analysis, as a quantitative research methodology, offers a comprehensive understanding of a specific field by synthesizing and quantitatively analyzing the literature. **Figure 1** shows the process of retrieving and filtering the records. In this study, CiteSpace and Excel 2019 software were used to conduct a systematic quantitative assessment and visualization analysis of key information, including annual publications, journals, authors, countries or institutions, highly cited literature, and keywords. A single node in the network map is used to represent various terms, such as authors, countries/regions, institutions, or keywords. The size of the node is proportional to the frequency of occurrence of the term, and the color of the node represents the temporal distribution of the term. The thickness of the connecting lines between the nodes indicates the strength of the relationship between the different terms. The centrality of a node serves as an important measure of its importance and influence in the co-occurrence network. Nodes with a centrality value greater than 0.1 are displayed in the atlas as purple circles, with the thickness of the circle proportional to the value of centrality (26). During the data processing, the following main parameters were set: (1) “Years Per Slice” was set to January 2010-March 2024, with one year designated as a time slice; (2) The node type was selected for analysis of authors, countries/regions, institutions, and keywords, respectively; (3) The selection criteria was set to g-index, k=25; (4) Other parameters were maintained at their default settings.

## 3 Results

### 3.1 Annual publication volume and trend

**Figure 2:**
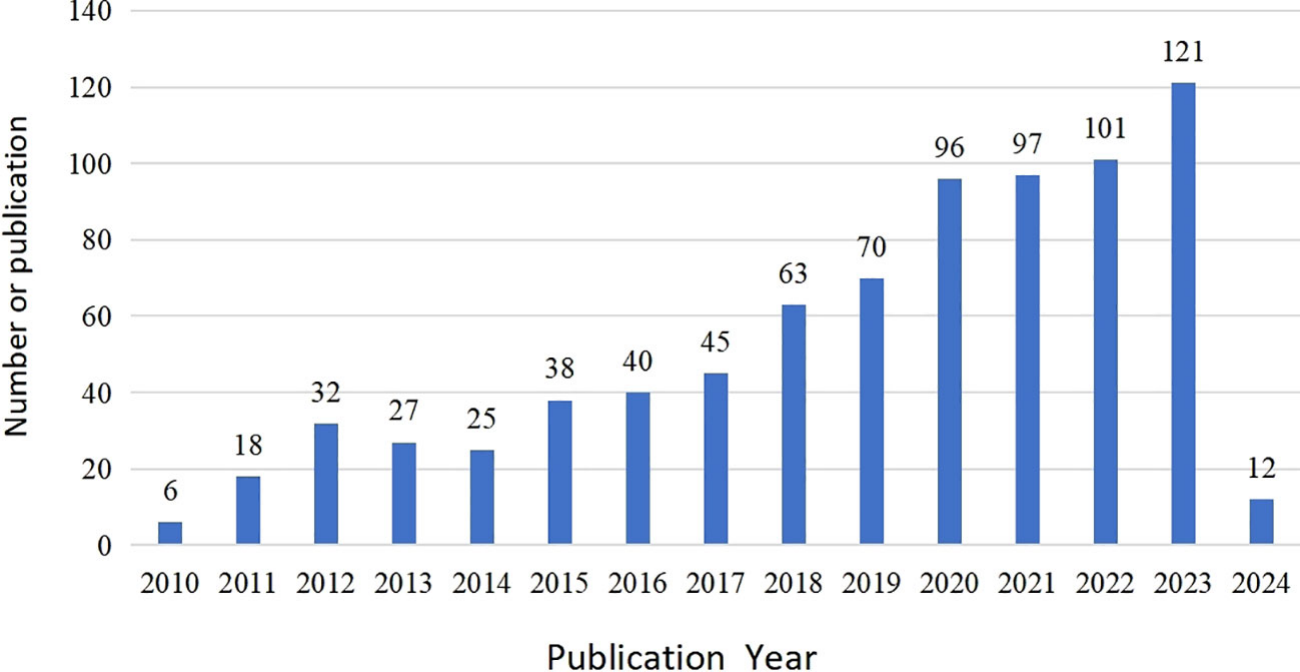
Annual publications.

Between 2010 and 2024, a total of 790 papers in the field of ACT for mental disorders were published. **Figure 2** provides a visual representation of the overall change in the annual number of publications. This change can be divided into three phases. The first phase, from 2010 to 2015, is characterized by an annual number of publications below 40. This period can be considered as the early stages of research in this field. The second phase runs from 2015 to 2017. During this period, the number of publications issued fluctuates slightly, but generally remains at a relatively low level, with the annual number of papers not exceeding 50. The third phase spans from 2018 to the present, during which the annual number of publications exceeded 50, exhibiting a consistent upward trend. In particular, the annual number of papers reached its highest point in 2023, with a total of 121 papers published. Over the past 15 years, the annual number of publications on ACT for mental disorders has demonstrated a significant and continuous growth trend, reflecting the increasing activity and depth of research in this field.

### 3.2 Analysis of journals

790 publications on the topic of ACT for mental disorders were published in 277 journals. Of these, 13 journals published more than 10 papers (34.89%), 101 journals published between 2 and 9 papers (44.76%), and the remaining 163 journals published only 1 paper (20.54%). In accordance with Bradford’s laws, in conjunction with the data from this study, the top 10 journals in terms of the number of articles published are designated as the core area journals. **Table 1** shows in detail the top 10 journals in terms of number of publications and number of citations, most of which are from the UK and the USA, with five from the USA and three from the UK. Journal of Contextual Behavioral Science is the journal with the highest number of publications with 94 articles, accounting for 11.884% of the total number of publications. It has a citation frequency of 1,157 times. Behavior Research and Therapy comes second with 27 articles, and although it does not have as many articles as the first journal, it is the most cited journal with 2,179 citations. Behavior Modification and Frontiers in Psychology were both in third place with 19 articles each, representing 2.4% of the total number of articles, with citation frequencies of 713 and 241 respectively. Of these 10 journals, 5 have an impact factor higher than 3.0. The average impact factor of these journals is 3.2, which shows the influence and recognition of these journals in the academic community.

**Table 1:** The top 10 most productive journals.

Rank	Journal	Records	Country	IF	Total citations	Average citations
1	Journal of Contextual Behavioral Science	94	Netherlands	5.0	1557	16.56
2	Behavior Research And Therapy	27	England	4.1	2179	80.7
3	Behavior Modification	19	USA	2.3	713	37.53
4	Frontiers In Psychology	19	Switzerland	3.8	241	12.68
5	BMJ Open	17	England	2.9	85	5
6	Clinical Case Studies	16	USA	1.0	75	4.69
7	Journal Of Pain	15	USA	4.0	863	57.53
8	Behavior Therapy	14	USA	3.7	585	41.79
9	Cognitive And Behavioral Practice	13	USA	2.9	446	34.31
10	Trials	12	England	2.5	139	11.58

IF, Impact Factor.

### 3.3 Analysis of the authors

**Figure 3:**
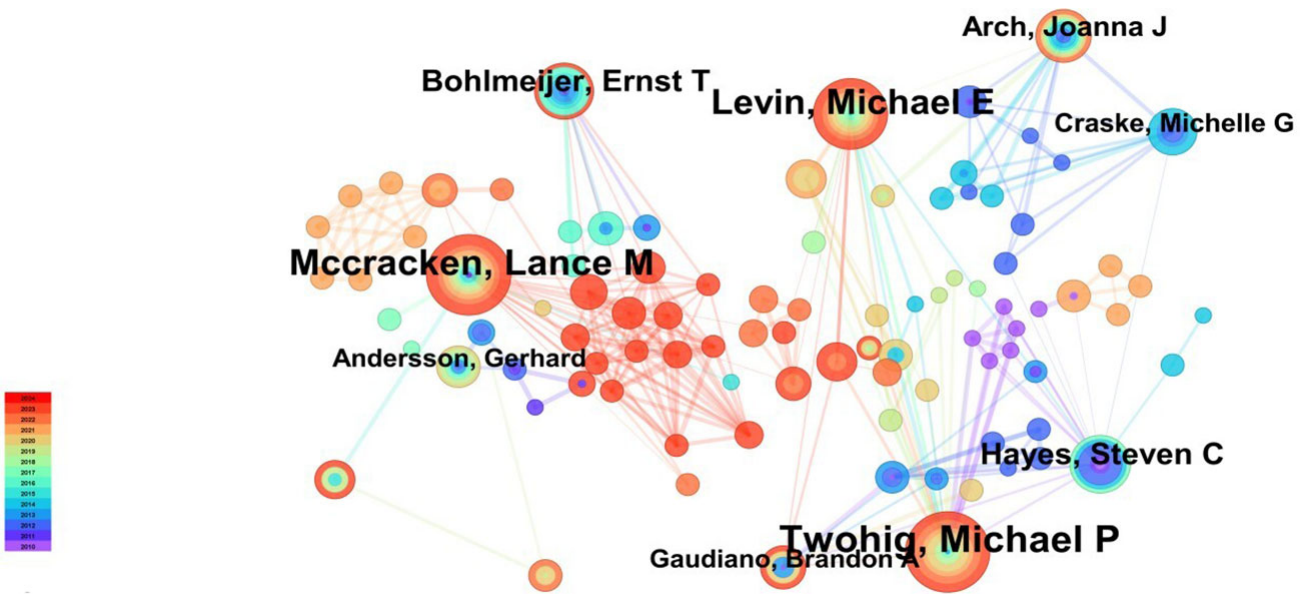
Cooperation network of authors.

3065 authors were involved in this study, with the top 10 authors publishing a total of 189 papers. These papers received a total of 9397 citations, accounting for 23.92% of the total number of publications and 43.94% of the total number of citations. In accordance with Lotka’s Law, the establishment of a stable core group of authors within a field is contingent upon the percentage of authors with one publication remaining below 60% (30). In the present study, the percentage of authors with one publication was 92.5% (2836/3065 ≈ 92.5%), well above the 60% threshold defined by Lotka’s Law. The extant data suggests that the current distribution of research authors in the field of ACT and mental disorders is fragmented and that a core group of authors has not yet been formed. **Table 2** shows the top 10 authors with the highest number of publications. The results indicate that the most productive author is Twohig MP (32 papers) from Utah State University, followed by McCracken LM (31 papers) from Uppsala University, Sweden, and Levin ME (23 papers) from Utah State University. To gain insight into author collaborations in the field, an author collaboration network analysis was performed using CiteSpace software, resulting in the creation of an author collaboration network map (**Figure 3**). The mapping reveals the formation of a dense and intricate network of collaborative relationships between researchers in the field. This indicates the extensive collaboration and profound interaction within the research field.

**Table 2:** The top 10 most productive authors.

Rank	Author	Record	Total citations	Average citations	H-index	Institutions
1	Twohig MP	32	1293	40.41	15	Utah State University
2	McCracken LM	31	1042	33.61	15	Uppsala University
3	Levin ME	23	1031	44.83	12	Utah State University
4	Bohlmeijer ET	19	2018	106.21	15	University of Twente
5	Hayes SC	17	1378	81.06	17	University of Nevada
6	Lappalainen R	16	393	24.65	9	University of Jyvaskyla
7	Gaudiano BA	14	221	15.79	8	Brown University
8	Arch JJ	13	818	62.92	10	University of Colorado Boulder
9	Craske MG	12	925	77.08	12	University of California Los Angeles
10	Scott W	12	278	23.17	9	King’s College London

### 3.4 Analysis of countries and institutions

**Figure 4:**
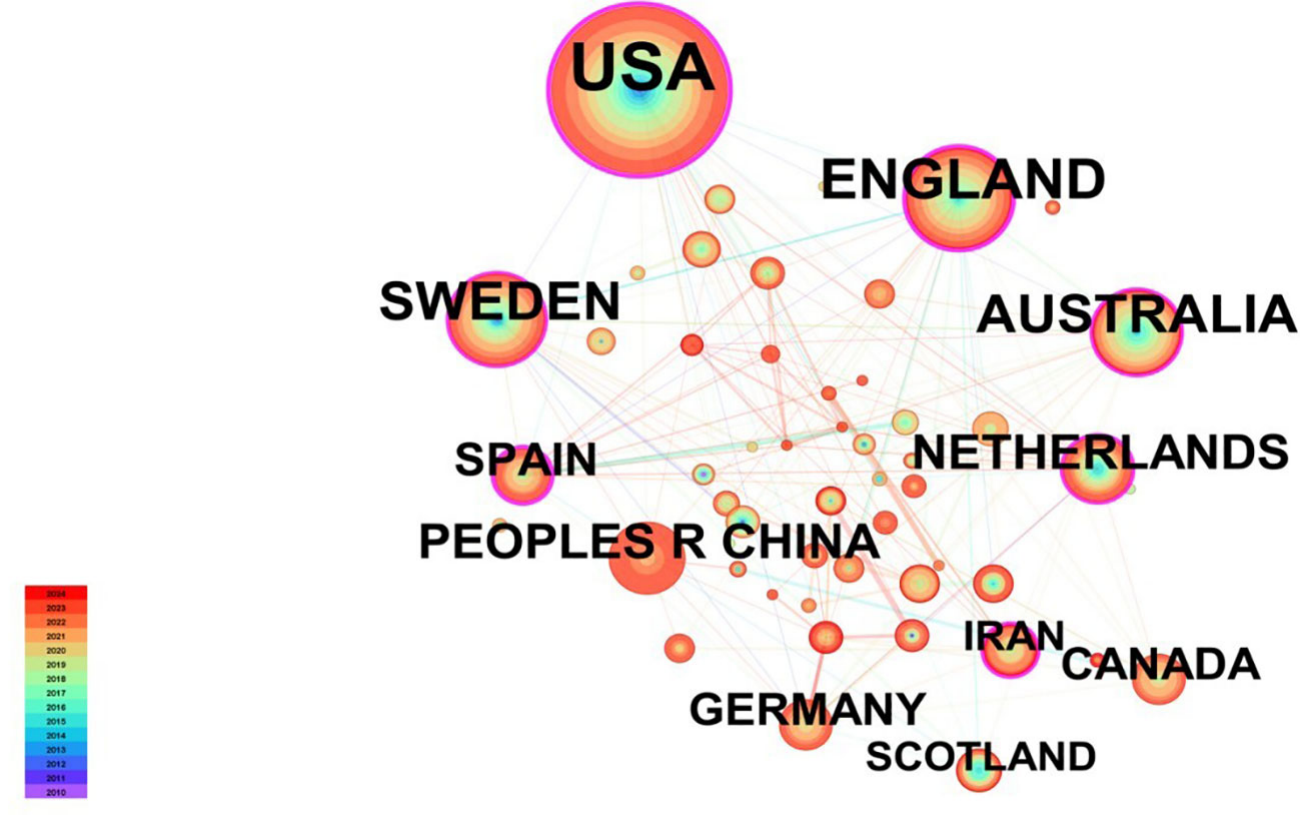
Cooperation network of countries.

A total of 52 countries are engaged in research on ACT for mental disorders. The ten countries with the highest number of publications are presented in **Table 3**. The country with the highest number of publications was the USA, with 283 publications, accounting for 35.78% of the total number of publications. Its number of publications was much higher than that of England (100 papers), which was in second place, and Sweden (82 papers), which was in third place. In the network mapping analysis, the top three centrality values are USA (0.49), Spain (0.24), and England (0.23), indicating that these countries exert a significant influence and centrality in the field. **Figure 4** shows the national collaborative co-occurrence network mapping, which comprises a total of 52 nodes (N), 170 connecting lines (E) and a network density of 0.1282. This suggests that approximately 790 articles were published by researchers from 52 countries and that 170 collaborations were established between these countries.

**Table 3:** The top 10 most productive countries.

Rank	Country	Record	Total citations	Average citations	Centrality
1	USA	283	8707	30.77	0.49
2	England	100	3239	32.29	0.23
3	Sweden	82	2802	34.17	0.18
4	Australia	78	2287	29.32	0.14
5	Netherlands	52	2913	56.37	0.12
6	China	51	376	7.37	0.04
7	Spain	40	982	24.55	0.24
8	Canada	39	1393	35.72	0.05
9	Germany	38	903	23.76	0.05
10	Iran	26	239	9.91	0.13

**Figure 5:**
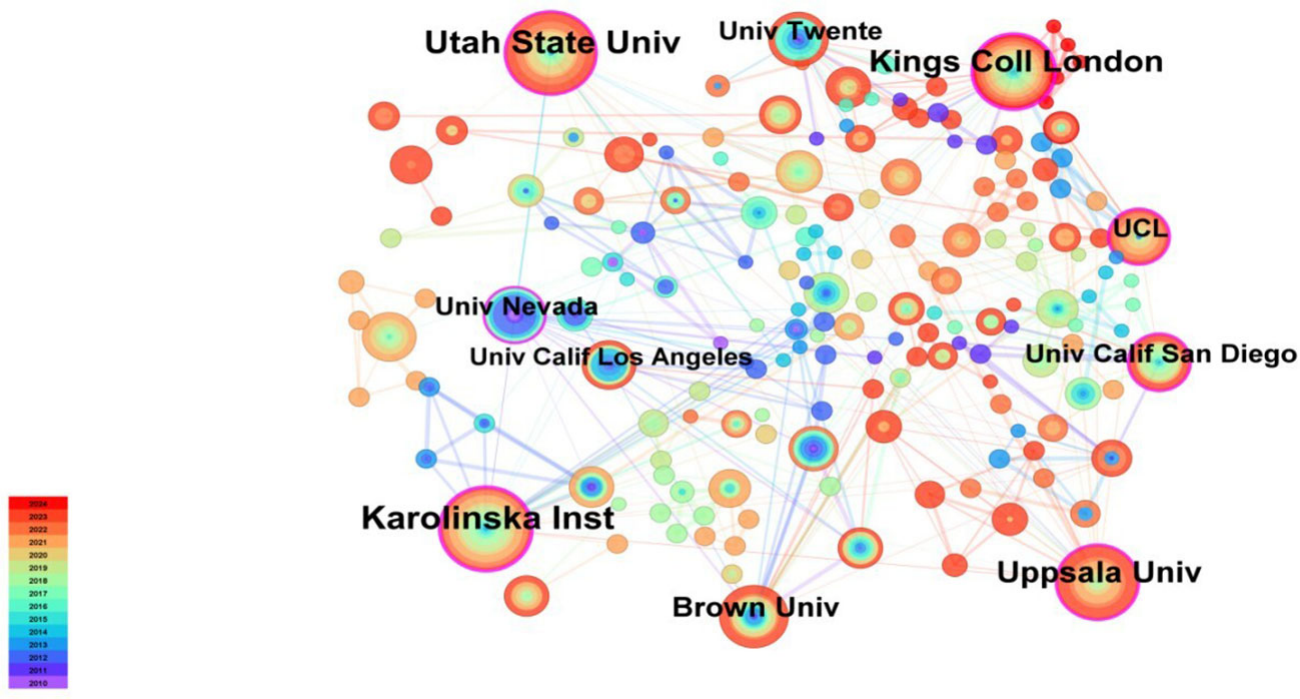
Cooperation network of institutions.

A total of 1,198 organizations were involved in research on ACT for mental disorders. **Table 4** presents the top 10 institutions with the highest number of publications. The institution with the highest number of publications was Karolinska Inst in Sweden (47 papers). Utah State University and King’s College London were the second and third most prolific institutions with 40 and 35 articles, respectively. Of the top 10 institutions publishing the most, five are based in the USA, demonstrating the country’s significant influence and research strength in the field of ACT for mental disorders. **Figure 5** shows the co-occurrence network of institutional collaborations. It can be seen that Karolinska Inst, the institution with the highest number of publications, has close collaborations with several institutions in the USA and the UK.

**Table 4:** The top 10 most productive institutions.

Rank	Institution	Record	Total citations	Average citations	Centrality
1	Karolinska Inst	47	2144	44.6	0.22
2	Utah State Univ	40	1578	35.86	0.1
3	Kings Coll London	35	1054	27.74	0.21
4	Uppsala Univ	33	639	16.82	0.10
5	Brown Univ	23	1080	30	0.07
6	Univ Calif San Diego	19	853	40.62	0.14
7	Univ Twente	18	2050	82	0.07
8	UCL	16	631	35.06	0.10
9	Univ Nevada	16	1739	86.95	0.11
10	Univ Calif Los Angeles	15	1150	57.5	0.01

### 3.5 High cited papers

A total of 21,384 citations were identified for 790 publications included, with an average of 27.03 citations per paper. **Table 5** provides a summary of the top 10 most cited papers. The most cited paper is Livingston, JD’s article in Addiction entitled “The effectiveness of interventions for reducing stigma related to substance use disorders: a systematic review” (501 citations). The second is A-Tjak, JGL’s article entitled “A Meta-Analysis of the Efficacy of Acceptance and Commitment Therapy for Clinically Relevant Mental and Physical Health Problems” (493 times), published in the journal Psychotherapy and Psychosomatics in 2015. The third most frequently cited article was that of Veehof, MM (2011) in the journal Pain, “Acceptance-based interventions for the treatment of chronic pain: a systematic review and meta-analysis” (463 times).

**Table 5:** The top 10 most cited articles.

Rank	Title	Total citations	Author	Year	Journal
1	The effectiveness of interventions for reducing stigma related to substance use disorders: a systematic review	501	Livingston, JD	2012	Addiction
2	A Meta-Analysis of the Efficacy of Acceptance and Commitment Therapy for Clinically Relevant Mental and Physical Health Problems	493	A-Tjak, JGL	2015	Psychotherapy And Psychosomatics
3	Acceptance-based interventions for the treatment of chronic pain: A systematic review and meta-analysis	463	Veehof, MM	2011	Pain
4	Acceptance- and mindfulness-based interventions for the treatment of chronic pain: a meta-analytic review	443	Veehof, MM	2016	Cognitive Behavior Therapy
5	Cognitive behavior therapy	345	Hughes, LS	2017	Clinical Journal Of Pain
6	A randomized, controlled trial of acceptance and commitment therapy and cognitive-behavioral therapy for chronic pain	333	Wetherell, JL	2011	Pain
7	Internet-delivered psychological treatments: from innovation to implementation	325	Andersson, G	2019	World Psychiatry
8	The efficacy of Acceptance and Commitment Therapy: An updated systematic review and meta-analysis	319	Öst, LG	2014	Behavior research And Therapy
9	Randomized Clinical Trial of Cognitive Behavioral Therapy (CBT) Versus Acceptance and Commitment Therapy (ACT) for Mixed Anxiety Disorders	245	Arch, JJ	2012	Journal Of Consulting And Clinical Psychology
10	A Randomized Clinical Trial of Acceptance and Commitment Therapy Versus Progressive Relaxation Training for Obsessive-Compulsive Disorder	226	Twohig, MP	2010	Journal Of Consulting And Clinical Psychology

### 3.6 Analysis of keywords

#### 3.6.1 Keyword co-occurrence analysis

**Figure 6:**
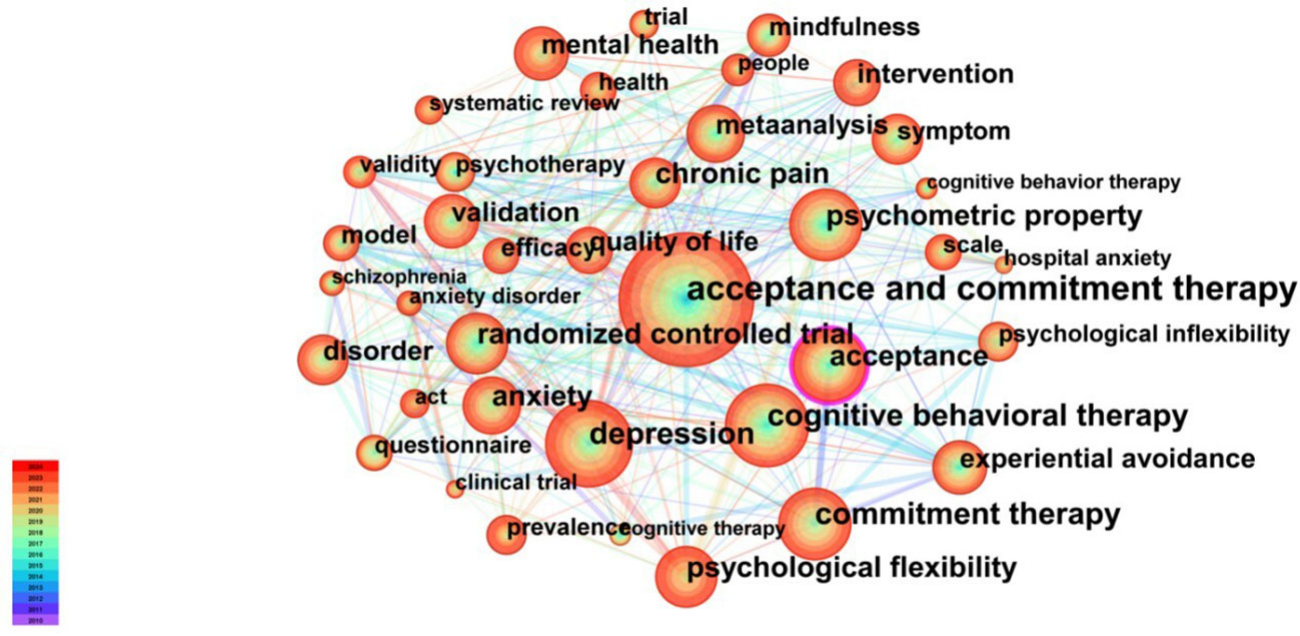
Cooccurrence network of keywords.

Keyword co-occurrence analysis reveals hotspots and trends in the field. A total of 38 significant keywords were identified based on the frequency of keyword occurrence. **Table 6** shows the top 20 most frequently occurring keywords. The top five keywords were acceptance and commitment therapy (440 times), cognitive behavioral therapy (440 times), depression (227 times), commitment therapy (207 times), and acceptance (207 times). The keyword co-occurrence network map (**Figure 6**) comprises a total of 497 nodes (N) and 3,618 connecting lines (E), with a network density of 0.0294. This indicates that there are 497 keywords involved and 3,618 pairs of keywords with direct co-occurrence relationships between them, forming the core research network in this field.

**Table 6:** The top 20 most frequent keywords.

Record	Keywords	Frequency	Centrality
1	acceptance and commitment therapy	440	0.02
2	depression	227	0.04
3	cognitive behavioral therapy	219	0.08
4	commitment therapy	207	0.04
5	acceptance	165	0.11
6	psychometric property	159	0.07
7	anxiety	139	0.04
8	randomized controlled trial	135	0.04
9	psychological flexibility	130	0.03
10	chronic pain	129	0.07
11	intervention	119	0.05
12	mental health	116	0.05
13	validation	114	0.05
14	quality of life	111	0.08
15	disorder	108	0.06
16	experiential avoidance	107	0.09
17	meta-analysis	98	0.03
18	mindfulness	90	0.03
19	symptom	80	0.03
20	efficacy	68	0.02

#### 3.6.2 Keyword clustering analysis

**Figure 7:**
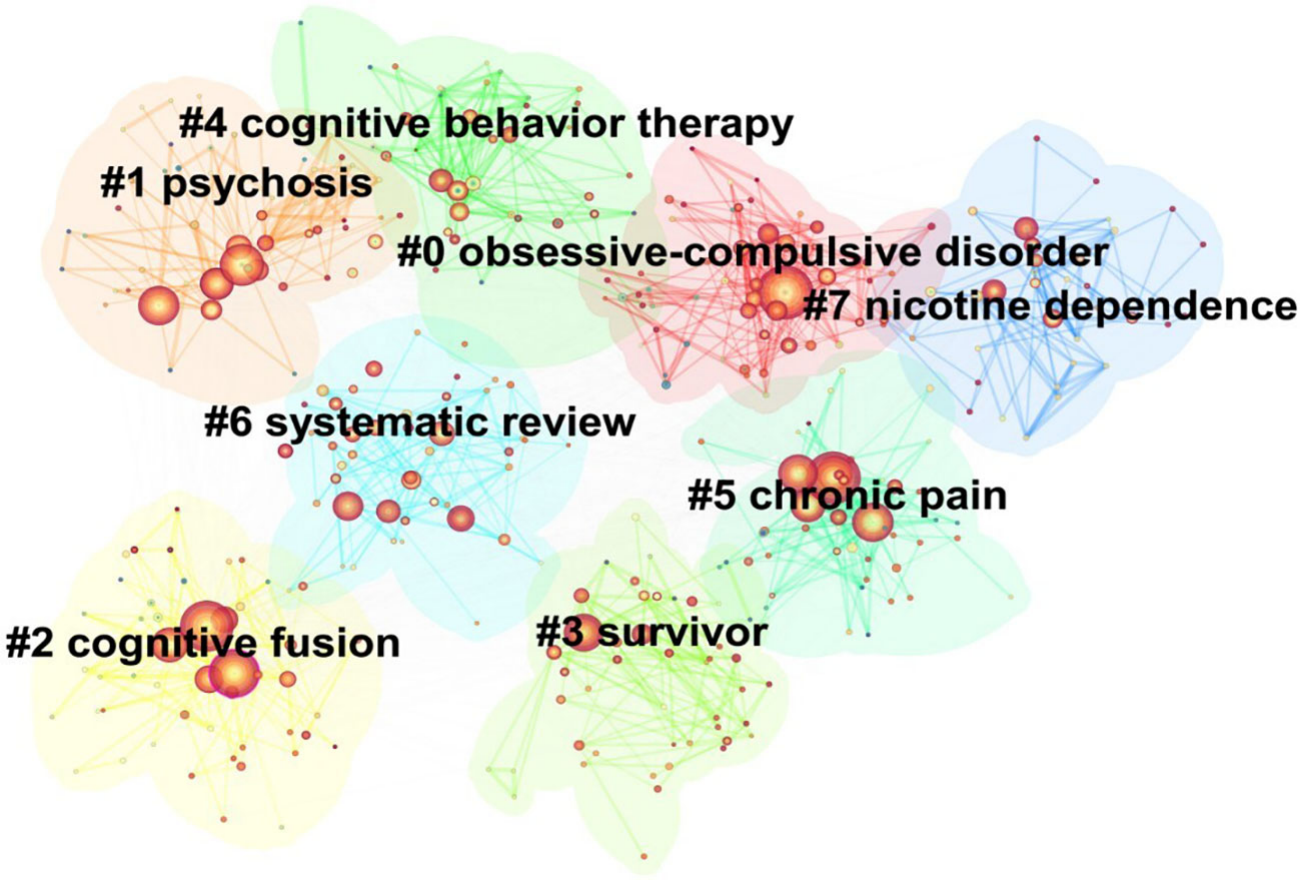
Cluster map of keywords.

Cluster analysis was employed to group the 497 keywords into eight main clusters. **Figure 7** shows the results of the cluster analysis, which are as follows: cluster #0 (obsessive-compulsive disorder), cluster #1 (psychosis), cluster #2 (cognitive fusion), cluster #3 (survivor), cluster #4 (cognitive behavior therapy), cluster #5 (chronic pain), cluster #6 (systematic review), and cluster #7 (nicotine dependence). The results showed that the keyword cluster exhibited a Q value of 0.3148 and an S value of 0.6528, indicating a significant cluster structure and high confidence in the clustering results.

#### 3.6.3 Keyword burst analysis

**Table 7** presents the top 25 keywords in the field with the highest burst intensity of occurrence. The ten keywords with the highest burst intensity were: values-based action, psychotherapy, panic disorder, cognitive behavior therapy, hospital anxiety, self-help, questionnaire, cognitive therapy, behavior therapy, and feasibility. Among the keywords, values-based action has the highest burst strength, indicating a significant increase in the frequency of this keyword in a short period of time. The keywords with the longest burst duration are values-based action (begin=2010, end=2016) and cognitive therapy (begin=2011, end=2017). At present, the terms systematic review, relational frame theory, and survivor are emerging as new hot topics in the current research landscape. The current research indicates a trend of becoming new hotspots, which portends new directions for future research.

**Table 7:** Top 25 keywords with the strongest citation bursts.

Keywords	Year	Strength	Begin	End	2010 - 2024
values based action	2010	6.88	2010	2016	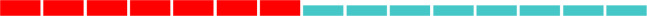
cognitive behavior therapy	2010	4.45	2010	2013	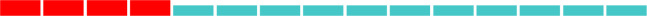
hospital anxiety	2010	4.36	2010	2015	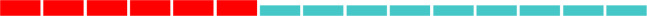
behavior therapy	2010	3.84	2010	2014	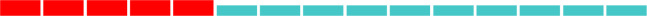
low back pain	2010	3.09	2010	2011	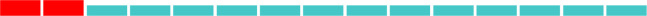
exposure	2010	2.6	2010	2015	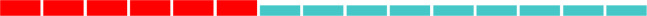
cognitive therapy	2011	4.02	2011	2017	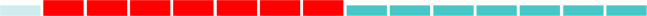
strategy	2011	2.85	2011	2014	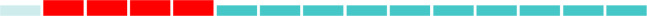
psychotherapy	2012	4.76	2012	2014	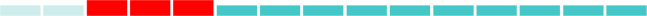
panic disorder	2012	4.75	2012	2014	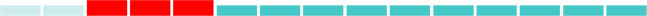
self help	2012	4.17	2012	2016	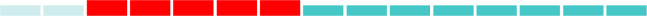
anxiety disorders	2012	3.5	2012	2017	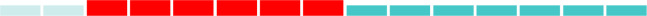
social phobia	2012	2.97	2012	2014	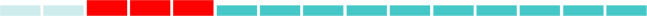
psychological treatment	2013	3.56	2013	2015	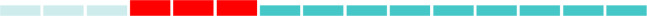
adherence	2013	3.05	2013	2016	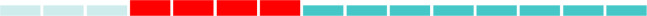
randomized trial	2013	2.93	2013	2015	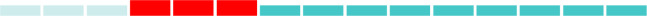
clinical significance	2014	2.69	2014	2017	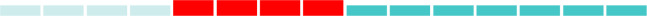
questionnaire	2012	4.05	2016	2018	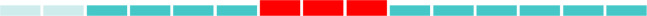
follow up	2016	3.5	2016	2018	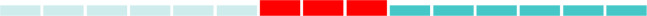
feasibility	2017	3.71	2017	2020	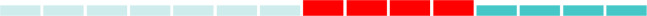
outcm	2015	2.6	2019	2021	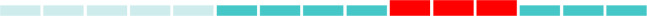
relational frame theory	2020	3.27	2020	2022	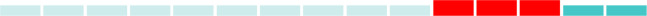 ;
systematic review	2017	2.92	2020	2022	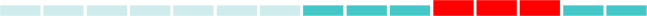
survivor	2021	2.9	2021	2024	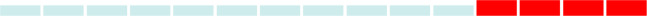
psychological intervention	2021	2.61	2021	2024	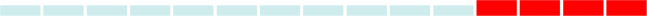

Coloured lines indicate the duration of keyword activity in academic research, specifically defined as follows: the starting point (left side) of each coloured line corresponds to the "Begin" year of the keyword (the starting time when it first became a research hotspot), and the endpoint (right side) corresponds to the "End" year of the keyword (the ending time when it ceased to be a research hotspot).

## 4 Discussion

In light of the findings presented above, this study will examine the current research trends, collaborations, and key research areas in the field of ACT and mental disorders. A bibliometric analysis of the literature on the topic of ACT for mental disorders published between 2010 and 2024 was conducted using the WOSCC database. A total of 790 publications were included, involving 3,065 authors from 1,198 institutions across 52 countries. These works were published in 277 journals, with a total of 21,384 citations, and an average of 27.03 citations per article.

The analysis of publication output reveals a gradual increase in global cooperation. Data spanning 15 years demonstrates a notable year-on-year increase in the number of publications. Since 2010, the initial volume of publications was low and although exhibited slight fluctuations in the following years, it remained low level overall. Since 2018, the upward trend in the number of articles has been particularly pronounced, reaching a peak in 2023. This may be associated with the notable rise in the incidence of mental disorders worldwide during the COVID-19 pandemic, which has resulted in a significant public health crisis and intensified the imperative for the implementation of psychotherapeutic interventions (31). As research has progressed, ACT has been developing and innovating in terms of treatment technology, intervention mode, and so forth. Furthermore, the theoretical system has been continuously expanded and improved. This has resulted in an increasing number of scholars dedicating their research efforts to this field. Journal distribution analysis reveals that most publications emanate from psychology or medical journals. Among the journals, the Journal of Contextual Behavioral Science is the most productive, with a total of 94 articles (11.88%) and 1,157 citations. The Behavior Research and Therapy journal is the most frequently cited among the top ten, with 27 articles (3.413%) and 2,179 citations. Finally, the journals Behavior Modification and Frontiers in Psychology, have 19 articles each, representing 2.4% of the total. The works have been cited 713 and 241 times. The representative journals in question are primarily focused on the fields of psychology, behavioral science and medicine, and are committed to the study of human behavior, psychological intervention processes and health interventions. In the future, ACT research will continue to expand and deepen the field of psychology and behavioral sciences, and will also continue to validate and enhance the relevant theoretical basis and intervention methods. As the medical field increasingly prioritizes the mental health of patients, the collaboration between ACT, and the medical field will be further enhanced. Although there has been a steady increase in the number of papers published on ACT for mental disorders over the past 15 years, the quality of the publications remains unsatisfactory. In particular, the proportion of papers published in high-level journals (with an impact factor of more than ten) is remarkably low. To some extent, this reflects the fact that research on current ACT findings in the field of mental disorders still requires further strengthening in terms of depth and academic impact. Researchers are encouraged to direct their efforts towards the investigation of cutting-edge issues, to undertake more targeted and forward-looking research, and to proactively submit research results that are both innovative and in-depth. It is imperative to enhance the probability of publication of research results in esteemed academic journals, with the ultimate objective of elevating the overall academic status of research in this field.

The number of publications by an author is often considered an important indicator of activity and contribution within a scientific field. Over the past fourteen years, numerous researchers have applied Acceptance and Commitment Therapy (ACT) within the realm of mental disorders, primarily forming teams centered around key figures such as Twohig MP, Mccracken LM, and Levin ME. Twohig MP (Utah State University) emerged as the most prolific contributor. He has made significant contributions to the field and has established productive collaborations with other researchers. The paper entitled “The effectiveness of interventions for reducing stigma related to substance use disorders: a systematic review” by Livingston, JD, was published in Addiction. The article had the highest total citation frequency (501 times). The review suggests that group-based ACT has been shown to be an effective approach for reducing self-stigma in individuals with substance use disorders. In addition, A-Tjak, JGL published a meta-analysis entitled “A Meta-Analysis of the Efficacy of Acceptance and Commitment Therapy for Clinically Relevant Mental and Physical Health Problems”. This demonstrates that Acceptance and Commitment Therapy is an efficacious approach for the treatment of anxiety, depression, addiction and somatic health conditions. In terms of analysis of country and institution, the USA has the highest number of publications on ACT for mental disorders. Furthermore, five of the top ten institutions in terms of publications are located in the USA. The USA has consistently ranked among the highest in the world in terms of publications (32). Government healthcare investment has significantly enhanced medical research productivity (33). The Karolinska Inst, in Sweden, has demonstrated the greatest output in this field over the past fifteen years, with 47 publications. This exemplifies the institution’s significant contribution to the field. A positive correlation has been observed between the high number of articles and the health expenditure of the United States and Sweden, as a proportion of their respective GDPs (32, 34). Our study also revealed that the countries and institutions contributing the most to the field are primarily located in developed countries, while developing countries contribute relatively less, possibly due to a lack of investment in mental health (9), reflecting the geographical differences in research contributions. This discrepancy in academic capacity may be closely related to the country’s level of development, economic strength, and research investment (35). Consequently, it is of paramount importance to promote the balanced distribution of scientific research resources through the allocation of funds, strengthen global research collaboration, and enhance equitable distribution of research resources in order to facilitate the advancement of the research field as a whole.

Keywords serve to reflect the core content of the paper to a certain extent and can be used as an important reference index for the analysis of research hotspots. The results of the keyword frequency analysis indicate this. Except for the keyword “acceptance and commitment therapy”, the other keywords with high frequency are focused on cognitive behavioral therapy (n=219), depression (n=227), anxiety (n=139), and randomized controlled trial (n=135). The high frequency of these keywords is indicative of the research focus and emerging trends in the field of mental disorders, specifically in the context of ACT.

CBT is a psychotherapeutic approach that originated in the 1960s for patients with psychiatric disorders, including depression, anxiety disorders, and somatoform disorders, among others. CBT is designed to effectively address a range of mental health issues caused by irrational cognitions (36). A number of studies have demonstrated (37) that CBT can correct cognitive biases, alleviate mood symptoms, and improve behavioral coping in patients with depressive disorders. Furthermore, CBT has been shown to be as effective as antidepressant medication alone in patients with mild to moderate depression. A randomized controlled trial (38) demonstrated that patients with somatoform disorders who underwent CBT exhibited significantly superior improvements in somatization symptoms and anxiety compared to those who received conventional treatment after a 24-week intervention. While CBT focuses on modifying patients’ cognitive processes and behavioral patterns, ACT, as an extension and development of CBT, focuses more on the relationship between people and their thoughts and feelings (17). A number of studies have compared the intervention effects of ACT and CBT. A randomized controlled trial of patients with anxiety disorders found that both ACT and CBT were significantly effective in reducing anxiety symptoms (39). However, ACT was found to be more prominent in increasing psychological flexibility, reducing experiential avoidance and facilitating patients’ values-based actions. Conversely, CBT was found to have a negative cognitive schema alteration advantage. In the context of depression treatment, a further study demonstrated the efficacy of both ACT and CBT in alleviating depressive symptoms (40). However, patients in the ACT group exhibited a more marked increase in life satisfaction following treatment. This phenomenon may be attributed to the fact that ACT facilitates patients in accepting their inner feelings and shifting their attention to real-life goals and values (41), thereby empowering them to lead a fulfilling life.

Depression is a psychiatric disorder characterized by persistent and significant depressed mood, accompanied by a variety of somatic conditions such as sleep disturbances, gastrointestinal disturbances, pain, and fatigue. In severe cases, it can lead to self-injury and suicidal behavior (42). According to the estimates of the WHO, by the year 2023 depression is set to become one of the leading causes of disability-adjusted life years within the global burden of disease (43). A meta-analysis of 18 studies involving 1,088 depressed patients demonstrated that ACT was an effective intervention for alleviating depressive symptoms, particularly among adults and those with mild depression (44). However, further in-depth studies are required for the underage group, patients with moderate to severe depression, and the long-term effects of treatment. In a comprehensive evaluation of ACT for depression (45), researchers included 15 studies. The results demonstrated that depressed patients receiving ACT interventions exhibited more stable mood states and greater psychological flexibility, with significant improvements in depressive symptoms. This finding that ACT may have a beneficial effect on the treatment of depression, particularly in specific patient groups. However, further studies on various patient groups and long-term effects are needed to comprehensively assess ACT’s effectiveness in depressed patients and support clinical practice with stronger evidence.

Anxiety disorders, as a prevalent psychiatric condition, are typified by feelings of tension, panic, worry, and fear, often accompanied by autonomic dysfunction (46). The high prevalence and high relapse rate of anxiety disorders have a significant impact on the quality of life of patients (47). A meta-analysis conducted by Burley et al. (48), the researchers included a total of nine studies for in-depth analysis. The main findings of this study were that following group therapy with ACT and during the subsequent follow-up period, adolescents with anxiety disorders experienced significant improvements in their anxiety symptoms. A further meta-analysis (49) demonstrated that ACT is an effective intervention for reducing anxiety symptoms in patients with a range of psychiatric and somatic disorders.

In summary, ACT occupies an important position in research in the field of mental disorders, and its close association with keywords such as cognitive behavioral therapy, depression, and anxiety disorders reflects the research hotspots and preoccupations in the field. In comparison with CBT, ACT has significant advantages in enhancing psychological flexibility, promoting values-based action and improving patient-specific life satisfaction. Conversely, CBT has been shown to be in modifying negative cognitive patterns. Although ACT has yielded promising outcomes in the treatment of prevalent mental health conditions such as depression and anxiety disorders, there is a paucity of research exploring its efficacy in other psychiatric disorders, including personality disorders, post-traumatic stress disorder (PTSD), and bipolar disorder. Future studies should aim to further explore the therapeutic effects of ACT in different patient groups and its long-term efficacy, in order to provide more solid evidence to support clinical practice. It is acknowledged that both ACT and CBT have their own unique advantages and application scenarios, and that the integration of the two can be considered when choosing a treatment. The extant literature has predominantly concentrated on the amelioration of symptoms by ACT, with a paucity of studies addressing the comprehensive effects on patients’ quality of life and social functioning. Consequently, subsequent studies should direct greater attention to the role of ACT in the comprehensive evaluation of patients’ functional recovery, with a view to ascertaining its clinical value.

The results of the keyword cluster map analysis indicate that obsessive-compulsive disorder (cluster #0) is a prominent disorder type in the field. OCD is a psychiatric disorder characterized by obsessive thoughts and compulsive behaviors (50), with a lifetime prevalence of 1% to 3% (51). A randomized controlled trial of 79 patients with OCD demonstrated that ACT was more effective in improving clinical symptoms in patients with OCD compared to relaxation therapy, and that patients showed higher adherence to ACT treatment (52). Nevertheless, the study’s limited sample size and the absence of long-term follow-up data impede the ability to ascertain the long-term efficacy of ACT in this patient population. The systematic review (clustering #6) has received considerable attention in the field and serves as an important research method. A systematic review is a comprehensive, systematic, objective, and quantitative method of literature review aimed at comprehensively analyzing and evaluating previous studies in the field (53). Within the hierarchy of evidence, systematic reviews and meta-analyses are frequently regarded as the highest level of evidence. A systematic review enables researchers to gain a deeper understanding of the current status and trends of research in a particular field, providing evidence for scientific research and clinical decision-making (54). As the clinical evidence for ACT continued to accrue, researchers initiated the integration of scattered studies through systematic evaluations. Han et al.’s (55) systematic review included 39 randomized controlled trials and found that ACT was effective in reducing anxiety, depression, and stress. Philip et al. (56) reviewed 16 papers and demonstrated that ACT significantly improved anxiety and depression levels in patients with OCD, especially when combined with medication. The systematic reviews provide compelling evidence for researchers to investigate the role of ACT in improving depressive symptoms, relieving anxiety, reducing stress, decreasing psychological distress, and enhancing quality of life in depth. Moreover, the reviews offer substantial evidence to support the development of clinical guidelines, signifying a significant transition from theoretical exploration to the evidence-based practice of ACT.

The results of the keyword burst analysis indicate that the term “values-based action” is the most frequently occurring keyword and has the longest duration of popularity in the short term. This finding reflects the increasing importance of ACT in the clinical application of patient values guidance. As one of the six core therapeutic processes of ACT, the concept of “values-based action” emphasizes the choice of action based on the individual’s core values and goals. Patients with mental disorders exhibit a range of cognitive, emotional, behavioral, and volitional impairments due to brain dysfunction (57). Such impairments frequently result in difficulties for patients in effectively expressing and realizing their values and goals in daily life. The ACT approach assists patients in aligning their core values with actionable goals, thereby fostering daily self-worth recognition that strengthens self-identity and life satisfaction. In the context of depression treatment, therapeutic practitioners have the capacity to enhance treatment adherence and recovery by guiding patients to clarify their personal values and develop action goals that are consistent with their core values (44). This finding indicates that clinical practitioners should prioritize the translation of the core therapeutic process of ACT into actionable, practical solutions. The term “cognitive therapy” is a keyword that has enjoyed equal popularity to “values-based action”, and occupies a significant role in the treatment of mental disorders. The objective of CT is to modify negative emotions and behaviors by modifying the patient’s maladaptive cognitions or faulty thinking. A meta-analysis of 15 randomized controlled trials (58) demonstrated that CT significantly reduced pathological worry in patients with generalized anxiety disorder. Furthermore, this treatment effect was maintained to a greater extent at subsequent follow-up, indicating that CT has a considerable therapeutic impact and sustained rehabilitation benefit for patients with psychiatric disorders, especially those with generalized anxiety disorder. This finding provides substantial support for clinical practice and patient rehabilitation. The sustained emergence of “self-help” (2012-2016) mirrors the development of the field of digital ACT interventions. This development is closely related to the initial integration of digital technologies into psychotherapy after 2010 (59). The advent of the Internet has led to a proliferation of digital self-help models, which have emerged as a pivotal avenue for exploring the enhancement of accessibility in ACT. A substantial number of studies have been undertaken between 2012 and 2016 to develop self-help ACT procedures based on web pages or basic applications (60–62). These studies sought to develop self-help ACT programs based on web pages or basic applications, with the objective of enabling users to complete psychological flexibility training autonomously from traditional therapeutic settings. This demonstrates that the self-help intervention model not only expands the application scenarios of ACT, but also solves the problems of traditional treatment through technological innovation. It is recommended that future research further exploits the potential of digital technologies to continuously optimize Internet-based self-service ACT procedures.

## 5 Theoretical and practical contributions

The present paper provides a theoretical foundation for the study of ACT in mental disorders. By means of a visual analysis of CiteSpace, the present study has systematically categorized and elucidated the core research themes of ACT in mental disorders, encompassing key areas such as “depression”, “anxiety”, “psychological flexibility”, and so forth. The study also identifies emerging hot topics such as “value-based action” and “digital intervention”. Keyword emergence analysis indicates that the terms “systematic review” and “relational framework theory” have grown exponentially in recent years, suggesting the necessity to delve deeper into the mechanisms and theoretical integration in the future. The paucity of international cooperation is highlighted, emphasizing the necessity for enhanced cross-regional collaboration to facilitate the optimal allocation and equitable distribution of research resources. Co-occurrence analyses of the high-frequency keywords “CBT” and “ACT” demonstrated that they are complementary in the treatment of mental disorders. In future research, the synergistic mechanism should be further explored to provide new perspectives for the theoretical development of third wave cognitive behavioral therapy.

The present paper provides a practical basis for the study of ACT in mental disorders. The study corroborates the substantial impact of ACT in ameliorating prevalent mental disorders, including depression and anxiety, while underscoring its efficacy in facilitating value-oriented action through the augmentation of psychological flexibility in patients. The present study provides an evidence base for clinicians to combine ACT with pharmacological treatments, CBT and other interventions. The advent of the keywords “self-help” and “web-based ACT” illuminates the pivotal role of digital technology in enhancing treatment accessibility. It is recommended that Internet-based ACT interventions be developed and combined with objective assessment tools, such as biomarkers, to optimize the effectiveness of the intervention. In view of the limited engagement of developing countries in research, it is proposed that assistance be provided to developing countries to enhance their research capacity through the promotion of enhanced international academic cooperation and exchange, with a view to promoting the balanced development and application of ACT on a global scale. Concurrently, the emphasis is being placed on the long-term impact of ACT on patients’ social function and quality of life. Furthermore, there is a proposal to establish a multi-dimensional efficacy assessment system.

## 6 Limitation

For the first time, this study systematically reviewed the relevant literature in the field of ACT for mental disorders based on the WOSCC database and revealed the research trends and frontiers in the field. However, there are still some limitations. Firstly, this study is based on CiteSpace software for bibliometric analysis, which is limited by the WOSCC database, and there is a possibility of data omission. Secondly, this study analyzes articles and reviews as the target publication types, with the language being restricted to English. This may result in a certain degree of limitation on the comprehensiveness and diversity of research in the field. Furthermore, the study was conducted based on “Topic” matter only, which may affect the comprehensiveness and completeness of the search results using this approach. In conducting the analysis of cited literature, only the frequently cited literature was given due consideration, while other relatively less cited literature was not afforded the same level of attention. This may have resulted in a bias in the analysis results in favor of the frequently cited literature, which in turn may have affected the accuracy of the judgement of this study on the research trends and frontier hotspots in the field of ACT and mental disorders.

## 7 Conclusion

The number of ACT research publications in the field of mental disorders has demonstrated a consistent and sustained increase over time. This finding is indicative of the sustained academic interest in the therapeutic potential of ACT in mental disorders, and suggests that research in this area is reaching a point of maturity. This study offers a more comprehensive analysis of the research trends and cutting-edge hotspots of ACT for mental disorders. From the perspective of research cooperation networks, although the core author group has not yet been established, there is a growing international dimension, indicating significant potential for development and dynamism within the field. However, the current low percentage of research results published in high-level journals suggests that there is still considerable scope for enhancing the depth and innovation of ACT in the field of mental disorders. Furthermore, through the utilization of keyword analysis, it was ascertained that ACT is closely associated with cognitive behavioral therapy, depression and anxiety. This not only elucidates the current research hotspots but also indicates the direction of focus for subsequent research. Further studies may combine ACT with other treatments, such as pharmacotherapy and CBT, to explore the potential for synergistic therapeutic effects in the field of mental disorders. In order to formulate a precise joint treatment plan according to the specific condition of the patient and individual differences, it is necessary to consider the interaction between the type and dose of drugs and the timing and frequency of ACT intervention when studying the combination of ACT and drug therapy. In combining with cognitive-behavioral therapy, the synergistic mechanism of the two therapies in improving patients’ cognition, behavior and psychological flexibility should be further explored, and the way of combining the two therapies in different stages of treatment should be clarified, so as to give full play to the advantages of each and improve the therapeutic effect. It is anticipated that such integrated studies will provide more comprehensive and effective treatment options for clinical practice. Concurrently, digital ACT intervention has been effectively developed in the domain of psychological intervention. The integration of digital technology and ACT has led to the emergence of a self-help intervention modality, which has broadened the scope of ACT’s application and addressed conventional treatment challenges. Future research should explore the potential of digital technology further, optimize Internet-based ACT procedures, and promote the new development of ACT in the field of mental disorders with the help of digital technology. The intervention is based on the core therapeutic process of ACT and is anchored in the patient’s committed actions, specifically value-based actions, with the aim of facilitating the patient’s psychological recovery and restoration of social functioning. Despite the gradual increase in the number of research studies investigating the effects of ACT for mental disorders, the quality of clinical research still requires improvement. Consequently, it is proposed that the efficacy, practicality, and replicability of ACT can be validated by implementing more rigorous and scientific research methodologies. In order to reduce the potential for bias and confounding factors to influence the results, studies employ rigorous randomized controlled trials or double-blind trials. This approach allows for a more accurate assessment of the effectiveness of ACT. In addition to the use of scales and questionnaires, objective assessment tools such as biological samples can be employed to gain a more comprehensive understanding of the effects of ACT on patients’ physiological and psychological states. In the case of complex or chronic psychiatric and psychological disorders, it is of paramount importance to consider the long-term efficacy and safety of ACT. In order to gain a more comprehensive understanding of the long-term impact of ACT on patients’ quality of life, it is essential to extend the follow-up period and collect more comprehensive data. As the efficacy of ACT in the treatment of mental disorders is further substantiated, policy makers may contemplate integrating it into the national healthcare system or public health policies with the objective of promoting this efficacious psychotherapeutic approach. The potential for ACT to be used in conjunction with other treatments suggests that researchers may be able to enhance the overall effectiveness of mental disorder treatment through interdisciplinary collaboration and integrative interventions. Finally, most of the countries and institutions that have made significant contributions in this area are located in developed countries, with geographical variations in research contributions. Through international academic exchanges and project cooperation, we can jointly promote the balanced development of ACT for mental disorders.

## Data Availability

The original contributions presented in the study are included in the article/supplementary material. Further inquiries can be directed to the corresponding author.
