# Correction: Research hotspots and frontiers of acceptance and commitment therapy for mental disorders: a bibliometric analysis

**DOI:** 10.3389/fpsyt.2025.1659792

**Published:** 2025-09-12

**Authors:** Xueqing Wang, Mingqi Wang, Huafang Li

**Affiliations:** ^1^ School of Nursing, Shandong Second Medical University, Weifang, China; ^2^ School of Physical Education, Shandong University, Jinan, China; ^3^ Department of Rehabilitation Medicine, Shandong Mental Health Center, Shandong University, Jinan, China

**Keywords:** acceptance and commitment therapy, mental disorders, hotspots, CiteSpace, bibliometric analysis

In the **Introduction**, the author name for citation 19 was incorrectly given as “Smith et al.” In the same paragraph, another citation was misspelled and the objective of their study incorrectly described as follows: “A randomized controlled trial by Kaipaine (20) et al. revealed that ACT significantly reduced depressive symptoms in nurses with depressive disorders by enhancing psychological flexibility, cognitive dissociation, and the clarification of values.”

A correction has been made to section **1 Introduction**, Paragraph 3:

“A systematic review conducted by Kelson et al. (19) demonstrated that web-based ACT is an effective and accessible treatment for patients with anxiety disorders, significantly improving the anxiety symptoms. A qualitative study by Kaipainen et al. (20) found that nurses reported through focus group interviews that mobile applications based on ACT can effectively assist in the care of depression and other mental health issues.”

The original version of this article has been updated.

